# The impact of SARS-CoV-2 (COVID-19) pandemic on educational and professional growth of young Italian epileptologists: a survey of the Young Epilepsy Section-Italian chapter

**DOI:** 10.1007/s10072-024-07836-7

**Published:** 2024-11-04

**Authors:** Silvia Masnada, Carlotta Spagnoli, Maddalena Duca, Daniela Chiarello, Tommaso Lo Barco, Bruna Nucera, On Behalf of Young Epilepsy Section—Italian chapter, Simona Balestrini, Luca De Palma, Giulia Battaglia, Lorenzo Ferri, Fedele Dono

**Affiliations:** 1 U.O.C. Neurologia Pediatrica, Buzzi Children’s Hospital, 20154 Milan, Italy; 2 S.O.C. Neuropsichiatria Infantile, Ospedale S. Maria Nuova, AUSL-IRCCS Di Reggio Emilia, 42123 Reggio Emilia, Italy; 3 U.O.C. Neuropsichiatria Infantile, Ospedale Civile Di Macerata, 62100 Macerata, Italy; 4 Neurology of Epilepsy and Movement Disorder Unit, Department of Neuroscience, Bambino Gesù Children’s Hospital, 00165 Rome, Italy; 5 Epilepsy Center, C. Poma Hospital, 46100 Mantua, Italy; 6 Department of Neurology, Hospital of Merano (SABES-ASDAA), Franz Tappeiner Hospital, Via Rossini, 5, 39012 Merano, Italy; 7 Paracelsus Medical University, 5020 Salzburg, Austria

**Keywords:** Italian epileptologists; Virtual systems; Educational and professional growth

## Abstract

**Objectives:**

In March 2020, the World Health Organization declared the coronavirus-related disease SARS-CoV-2 infection pandemic. Italy was one of the most affected countries and managed the emergency also by a health care reorganization.

**Methods:**

The Education and Career Development Task Force of the Young Epilepsy Section-Italy (YES-I) designed a survey to assess the impact of the pandemic on the training and work of young epileptologists (< 40 years).

**Results:**

Fifty-three responses were collected: 45.3% were resident, 9.4% PhD students and the remainder specialists. Clinical activity changed for most (83%) during the pandemic. Educational activity at epilepsy centers was reduced for 35.8% of the survey participants, while 30.2% of research projects involving patients participation were stopped to switch mainly to COVID-19-related research. For 73.6% of survey participants, attending online courses and congresses was easier in terms of cost and organization, although for 50.9% the level of training was lower in quality. In contrast, 58.5% rated the webinars organized by YES-I very educational. Less than 50% of the clinicians used telemedicine in the pandemic period and continue to use it. Despite several positive aspects of virtual medicine, a small number (32.1%) of our interviewees were satisfied from telemedicine and few of them (30.2%) reported that it led to improvement of clinical practice.

**Conclusions:**

Our survey showed that the pandemic has had a negative impact on training, research and clinical activity in the epilepsy field; moreover, it underlined the critical aspects of virtual communication methods in order to improve its use for the future.

## Introduction

In March 2020, the World Health Organization declared the SARS-CoV-2 (Severe Acute Respiratory Syndrome Coronavirus 2)/Coronavirus Disease 19 COVID-19) infection pandemic (https://www.who.int/europe/emergencies/situations/covid-19). Italy was one of the most damaged European Countries [1]. During the pandemic period, institutions have established precise rules in order to reduce social contacts; the Italian Health Care Minister has granted permission for individuals to leave their residences exclusively for the purposes of employment, medical appointments, or the procurement of essential sustenance, subject to specific regulatory (https://www.gazzettaufficiale.it/eli/id/2020/03/08/20A01522/sg). Institutions encouraged people to reach hospitals only when strictly necessary, in order to reduce contact with infected people, and the health care system had to be completely reorganized [1]. Clinicians faced a downfall of outpatient visits in parallel with the need to maintain close contact with patients to guarantee the required management and treatment indications [2, 3]. In the epilepsy field, epileptologists need to have frequent contact with patients, particularly those with drug-resistant epilepsy in order to manage seizures recurrence and adverse events.

On the other side, epileptologists, both trainees and consultants in pediatric and adult departments, were required to provide care for COVID-19 patients [2], resulting in a difficulty of facing the challenge of continuing educational and research programs in epilepsy, having meetings and congresses to increase their professional growth and knowledge [4]. In this scenario, telemedicine provided the opportunity to establish a remote interface between healthcare facilities and patients, whether with or without video capability, and as such, it was implemented by numerous healthcare centers. Remote systems also allowed consultants and trainees to attend meetings, conferences and educational programs, to possibly mitigate the psychological impact of that devastating reduction of social contact. Remote communications had both positive and negative consequences on research, educational, and clinical activity [4, 5]. Moreover, remote systems helped clinicians and patients in improving the wide psychological consequences and lifestyles changes of the pandemic [6, 7]. The Italian Section of the Young Epilepsy Section of International League Against Epilepsy (ILAE-YES), i.e. Young Epilepsy Section-Italian (YES-I), (https://www.lice.it/LICE_ita/YES-I/YES_I.php) was developed in 2019 within the Italian League Against Epilepsy (LICE), (https://www.ilae.org/about-ilae/structure-and-working-groups/commissions-and-sections/young-epilepsy-section-yes), aiming to improve professional development opportunities and education. The Education and Career Development Task Force of YES-I is dedicated to promote education through training experiences in Italy and abroad for students, residents and young consultants (up to 40 years of age), dissemination of job opportunities or doctoral fellowships, and organization of educational meetings, which continued during the pandemic period in virtual mode (https://www.lice.it/LICE_ita/YES-I/YES_I.php ).

Given the reorganization of the health care system during the pandemic, the YES-I Education and Career Development Task Force launched a remote survey with the aim of assessing the impact of the COVID-19 pandemic on professional, educational and research activities of Italian students, trainees and young consultants.

## Materials and methods

The electronic survey was drafted by the Education and Career Development Task Force, YES-I, with support from YES executive committee members.

The survey consisted of eighteen multiple choice questions, and was divided into three sections: a first part concerning demographic data (gender, age, geographical area, current working position, interest in adult versus pediatric patients—five questions), a second part aimed to investigate changes in educational and research programs of trainees during Sars-Cov2 pandemic (six questions), and a third part focused on work changes and the use of telemedicine during the pandemic period (seven questions) (all the questions and results of the survey are listed in Table 1). The survey was part of a broader questionnaire that included other aspects involving young people such as gender inequality in epileptology [8] and educational and career needs [9].

**Table 1:** Questions and results of the survey listed: Demographic data, Pandemic changes on educational and research activities, Impact of online courses and conferences (including YES webinars) on education, Pandemic changes on work, Telemedicine

Demographic data			
	Female n (%)	Male n (%)	Total n (%)
Gender:	39/53 (73.6%)	14/53 (26.4%)	53/53 (100%)
Age:			
20–25	1/39 (2.6%)	1/14 (7.1%)	2/53 (3.8%)
25–30	13/39 (33.3%)	7/14 (50.0%)	20/53 (37.7%)
30–35	12/39 (30.8%)	2/14 (14.3%)	14/53 (26.4%)
> 35	13/39 (33.3%)	4/14 (28.6%)	17/53 (32.1%)
Geographical area:			
North	23/39 (59.0%)	8/14 (57.1%)	31/53 (58.5%)
Centre	8/39 (20.5%)	2/14 (14.3%)	10/53 (18.9%)
South	5/39 (12.8%)	1/14 (7.1%)	6/53 (11.3%)
Major islands	3/39 (7.7%)	3/14 (21.4%)	6/53 (11.3%)
Current professional position:			
Student	0	0	0
Trainee	16/39 (41.0%)	8/14 (57.1%)	24/53 (45.3%)
Consultant	20/39 (51.3%)	4/14 (28.6%)	24/53 (45.3%)
PhD student/Fellow	3/39 (7.7%)	2/14 (14.3%)	5/53 (9.4%)
Other	0	0	0
Age group of patients cared for:			
Children	25/39 (64.1%)	1/14 (7.1%)	26/53 (49%)
Adults	12/39 (30.8%)	12/14 (85.7%)	24/53 (45.3%)
Both	2/39 (5.1%)	1/14 (7.1%)	3/53 (5.7%)
Pandemic changes on educational and research activities			
Did the pandemic change educational activities?			
Not at all	6/39 (15.4%)	2/14 (14.3%)	8/53 (15.1%)
Partial involvement in COVID-19 wards	7/39 (18.0%)	4/14 (28.6%)	11/53 (20.7%)
Indirect involvement in the management of COVID-19 patients (e.g. consultations to other units)	13/39 (33.3%)	3/14 (21.4%)	16/53 (30.2%)
Activities completely changed due to the pandemic	7/39 (18.0%)	2/14 (14.3%)	9/53 (17.0%)
The responder is not in training	6/39 (15.4%)	3/14 (21.4%)	9/53 (17.0%)
The pandemic has mainly changed the educational activities because of:			
Reduction or cancellation of rotations in affiliated structures part of the educational network	5/39 (12.8%)	5/14 (35.7%)	10/53 (18.9%)
Impossibility to carry out an educational experience at specialized centers abroad	5/39 (12.8%)	1/14 (7.1%)	6/53 (11.3%)
Impossibility to carry out an educational experience at specialized centers in Italy	1/39 (2.6%)	1/14 (7.1%)	2/53 (3.8%)
Reduction of educational activities at their own epilepsy center	15/39 (38.5%)	4/14 (28.6%)	19/53 (35.8%)
No change in educational activities	10/39 (25.7%)	3/14 (21.4%)	13/53 (24.5%)
Educational activities have improved	3/39 (7.7%)	0	3/53 (5.7%)
How did the research activities change during the pandemic?			
No research activities performed before, during, or after	3/39 (7.7%)	2/14 (14.3%)	5/53 (9.4%)
Interruption of projects involving neurological and non-neurological patients	14/39 (35.9%)	2/14 (14.3%)	16/53 (30.2%)
Only projects on COVID19-related topics were performed	4/39 (10.3%)	5/14 (35.7%)	9/53 (17.0%)
No changes	16/39 (41.0%)	3/14 (21.4%)	19/53 (35.8%)
Increase in all research areas	2/39 (5.1%)	2/14 (14.3%)	4/53 (7.5%)
Impact of online courses and conferences (including YES webinars) on education			
How conducting courses and conferences online has affected your professional education/growth?			
It was easier to take part to online activities due to reduced costs	11/39 (28.2%)	7/14 (50.0%)	18/53 (34.0%)
It was less difficult to take part to online activities for work organization	18/39 (46.1%)	3/14 (21.4%)	21/53 (39.6%)
It was more difficult to take part to online activities for work organization	6/39 (15.4%)	3/14 (21.4%)	9/53 (17.0%)
It was encouraged by the training school compared to on-site events	3/39 (7.7%)	1/14 (7.1%)	4/53 (7.5%)
No impact	1/39 (2.6%)	0	1/53 (1.9%)
Did participation to online courses and conferences affect the quality of education?			
Not at all	5/39 (12.8%)	1/14 (7.1%)	6/53 (11.3%)
Lower quality was observed	18/39 (46.1%)	9/14 (64.3%)	27/53 (50.9%)
No differences were observed compared to on-site events	15/39 (38.5%)	4/14 (28.6%)	19/53 (35.8%)
The educational level was improved compared to on-site events	1/39 (2.6%)	0	1/53 (1.9%)
How would you rate the educational content of YES-I webinars?			
Quite educational	8/39 (20.5%)	1/14 (7.1%)	9/53 (17.0%)
Very educational	21/39 (53.8%)	10/14 (71.4%)	31/53 (58.5%)
I did not join any YES-I webinars	10/39 (25.7%)	3/14 (21.4%)	13/53 (24.5%)
Pandemic changes on work			
Did the pandemic change your work activity?			
Not at all	7/39 (18.0%)	2/14 (14.3%)	9/53 (17.0%)
Partial involvement in COVID-19 wards	11/39 (28.2%)	5/14 (35.7%)	16/53 (30.2%)
Indirect involvement in the management of COVID-19 patients (e.g. consultancies to other units)	16/39 (41.0%)	5/14 (35.7%)	21/53 (39.6%)
Activities completely changed due to the pandemic	5/39 (12.8%)	2/14 (14.3%)	7/53 (13.2%)
How did the pandemic change the way you work?			
No change	3/39 (7.7%)	2/14 (14.3%)	5/53 (9.4%)
Reduction of specific work activities with a shift towards other disciplines in medical areas	11/39 (28.2%)	8/14 (57.1%)	19/53 (35.8%)
Increase of specific work activities (i.e. clinical and/or neurophysiological tests)	5/39 (12.8%)	1/14 (7.1%)	6/53 (11.3%)
Increase of telemedicine	19/39 (48.7%)	2/14 (14.3%)	21/53 (39.6%)
Other	1/39 (2.6%)	1/14 (7.1%)	2/53 (3.8%)
Telemedicine			
Did your center adopt telemedicine during COVID-19 pandemic?			
Adopted only during the pandemic	15/39 (38.5%)	5/14 (35.7%)	20/53 (37.7%)
Never adopted	3/39 (7.7%)	3/14 (21.4%)	6/53 (11.3%)
Adopted during the pandemic and after	18/39 (46.1%)	5/14 (35.7%)	23/53 (43.4%)
None of the above	3/39 (7.7%)	1/14 (7.1%)	4/53 (7.5%)
The use of telemedicine consisted in:			
Communication through institutional e-mail	6/39 (15.4%)	1/14 (7.1%)	7/53 (13.2%)
Communication through personal e-mail	1/39 (2.6%)	2/14 (14.3%)	3/53 (5.7%)
Telephone consultations/clinics	5/39 (12.8%)	5/14 (35.7%)	10/53 (18.9%)
Other channels (institutional dedicated softwares or video calls)	22/39 (56.4%)	5/14 (35.7%)	27/53 (50.9%)
Other	5/39 (12.8%)	1/14 (7.1%)	6/53 (11.3%)
How did telemedicine change the way you worked?			
No changes	14/39 (35.9%)	5/14 (35.7%)	19/53 (35.8%)
Worsening of working activities	7/39 (18.0%)	4/14 (28.6%)	11/53 (20.7%)
Improvement of working activities	12/39 (30.8%)	5/14 (35.7%)	17/53 (32.1%)
Other	6/39 (15.4%)	0	6/53 (11.3%)
How did telemedicine change the relationship with the patients?			
No changes	15/39 (38.5%)	3/14 (21.4%)	18/53 (34.0%)
Worsening of the relationship	10/39 (25.7%)	7/14 (50.0%)	17/53 (32.1%)
Improvement of the relationship	9/39 (23.0%)	3/14 (21.4%)	12/53 (22.6%)
Other	5/39 (12.8%)	1/14 (7.1%)	6/53 (11.3%)
Did telemedicine impact on your professional growth?			
Not at all	12/39 (30.8%)	2/14 (14.3%)	14/53 (26.4%)
A little	16/39 (41.0%)	7/14 (50.0%)	23/53 (43.4%)
To some extent	11/39 (28.2%)	5/14 (35.7%)	16/53 (30.2%)
A lot	0/39	0/39	0/39

The survey was addressed to Italian neurology trainees and consultants working in the epilepsy field. It was disseminated by a QR code during the 43rd National LICE Congress held in Padua, 8–10 June 2022 and distributed by email to all the LICE members up to 40 years of age until the 7th of September 2022.

Before starting the survey, the respondents were asked for consent to use their personal data for scientific purpose and publication.

Data from the survey were processed using Microsoft Excel. We tabulated the results for each question. A descriptive analysis, calculating absolute numbers and percentages for each reply, were performed.

## Results

### General results

Fifty-three completed surveys were collected, 73.6% by females and 26.4% by males; 58.5% of our survey participants were ≥ 30 years of age (26.4% ≥ 30–35 years, 32.1% ≥ 35–40), 41.5% were 20 to 30 years; the number of responders represents a response rate of 9.6% within the entire cohort of LICE members aged up to 40 years. Most of the responders (58.5%) work in the North of Italy, whilst 18.9% were from the Center and 11.3% from the South and the Islands.

Considering the professional activity, 45.3% of those who participated to the survey were residents, 9.4% PhD students, and the remaining were consultants working in the epilepsy field; 49% of them in the pediatric and 45.3% in the adult field.

The results from both parts of the survey are presented in Table 1.

### Changes to educational and research programs during the COVID-19 pandemic period

**Fig. 1:**
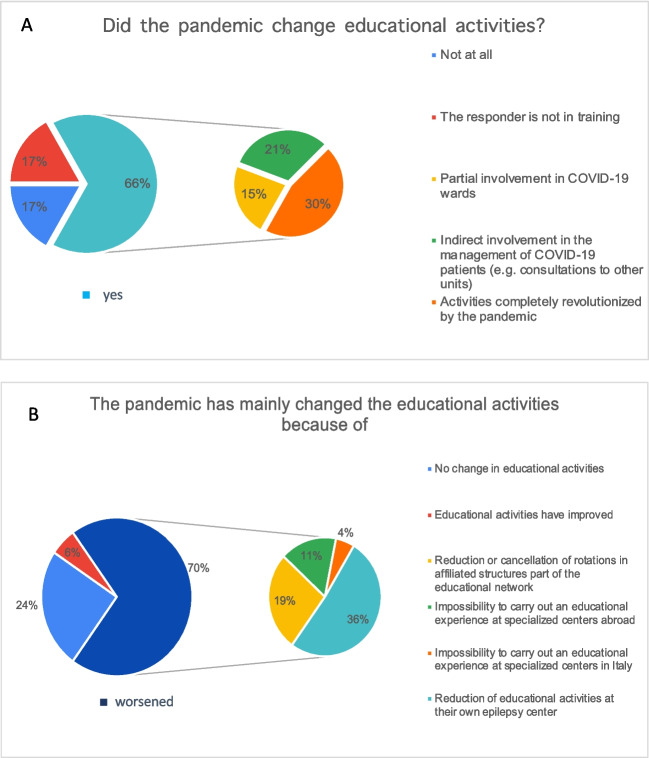
Changes to educational programs during the COVID-19 pandemic period. Figure shows the changes during the pandemic period on educational activities (**A**, **B**)

Sixty-eight percent of the trainees reported a reorganization of their activities, mainly related to the management of patients affected by SARS-CoV-2. Some (20.7%) survey participants were directly involved with care of COVID-19 patients whilst others (30.2%) reported less direct involvement (e.g. clinical consultations in COVID-19 departments due to neurological problems of infected patients). For 15.1% of trainees, educational activities remained unchanged. 17% of the responders did not express their opinion at this specific question because they are consultant not anymore involved in educational activities. So, only for 15.1% of the trainees, educational activities remained unchanged during the pandemic period. Educational programs were reduced in University Hospitals (35.8%) and in affiliated hospitals that were part of the training network (18.9%). Training programs in national third-level Epilepsy Centers (11.3%) and abroad (3.8%) were not allowed because of the pandemic (Fig. 1A, B). No substantial differences were observed among respondents from various regions of Italy (Table 2).

**Table 2:** Selected questions and results of the survey listed with the aim of evaluate regionals differences

Pandemic changes on educational and research activities						
	**North n (%)**		**Center n (%)**		**Sud n (%)**	**Islands n (%)**
The pandemic has mainly changed the educational activities because of:						
Reduction or cancellation of rotations in affiliated structures part of the educational network	4/31 (12.9%)		2/10 (20%)		1/6 (16.6%)	3/6 (50%)
Impossibility to carry out an educational experience at specialized centers abroad	4/31 (12.9%)		1/10 (10%)		0	1/6 (16.6%)
Impossibility to carry out an educational experience at specialized centers in Italy	1/31 (3.2%)		0		1/6 (16.6%)	0
Reduction of educational activities at their own epilepsy center	11/31 (35.4%)		5/10 (50%)		2/6 (33.3%)	1/6 (16.6%)
No change in educational activities	9/31 (29%)		1/10 (10%)		2/6 (33.3%)	1/6 (16.6%)
Educational activities have improved	2/31 (6.4%)		1/10 (10%)		0	0
Impact of online courses and conferences on education						
Did participation to online courses and conferences affect the quality of education? Not at all Lower quality was observed No differences were observed compared to on-site events The educational level was improved compared to on-site events	1/31 (3.2%) 15/31 (48.3%) 14/31 (45.1%) 1/31 (3.2%) 0			2/10 (20%) 5/10 (50%) 3/10 (30%) 0 0	3/6 (50%) 2/6 (33.3%) 1/6 (16.6%) 0 0	0 5/6 (83.3%) 1/6 (16.6%) 0 0
Telemedicine						
Did your center adopt telemedicine during COVID-19 pandemic? Adopted only during the pandemic Never adopted Adopted during the pandemic and after None of the above	13/31 (41.9%) 2/31 (6.4%) 13/31 (41.9%) 3/31 (9.6%) 0	2/10 (20%) 1/10 (10%) 6/10 (60%) 1/10 (10%) 0			1/6 (16.6%) 2/6 (33.3%) 3/6 (50%) 0 0	4/6 (66.6%) 1/6 (16.6%) 1/6 (16.6%) 0 0

**Fig. 2:**
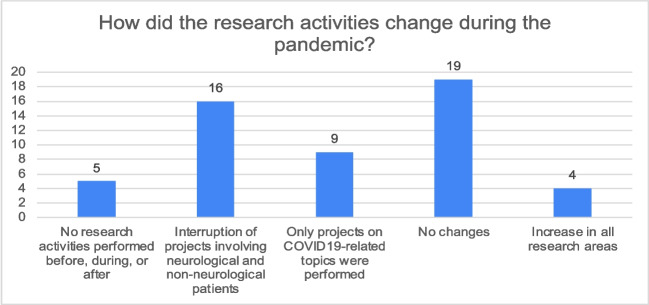
Changes to research programs during the COVID-19 pandemic period. Figure shows the changes during the pandemic period on research

The 54.7% of respondents reported a change in research activities during the pandemic period: research projects involving patient participation were interrupted in 30.2%, while COVID-19-related research continued for 17% (Fig. 2).

**Fig. 3:**
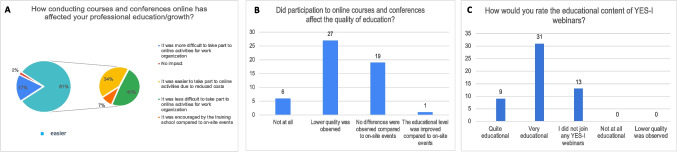
Impact of online educational programs during the COVID-19 pandemic period. Figure shows the impact of online conferences and congress on educational growth (**A**), the quality of online education (**B**) and, in details, the quality of webinars organized by YES-I (**C**)

According to 81% of the survey participants, when compared to in person courses and congress, online teaching was easier in terms of cost (34%) and work organization (39.6%). The educational level was considered poorer by about half of participants (50.9%), while only 1.9% reported an improvement of educational level; however, when the data was analyzed considering each Italian regions, respondents from the North, Center, and Islands predominantly rated the quality of online courses as low, with a slightly smaller percentage indicating no differences. In contrast, in the South, the majority of respondents (50%) did not perceive any differences in quality between online and in-person education, while 33% reported a lower quality for online courses. Globally, the online training organized by the Italian Young Epilepsy Section in order to compensate the reduction of educational activities, was considered a high-quality training experience (58.5%) (Fig. 3A, B, C).

### Work changes and use of telemedicine during the COVID-19 pandemic

Among the survey participants, a significant proportion (83%) reported changes in clinical activity within their Epilepsy Centers, with a shifting from their specialized roles to directly manage of SARS-CoV-2 patients, or indirectly contributing to patient care, such as providing consultation for neurological issues in COVID-19 patients.

**Fig. 4:**
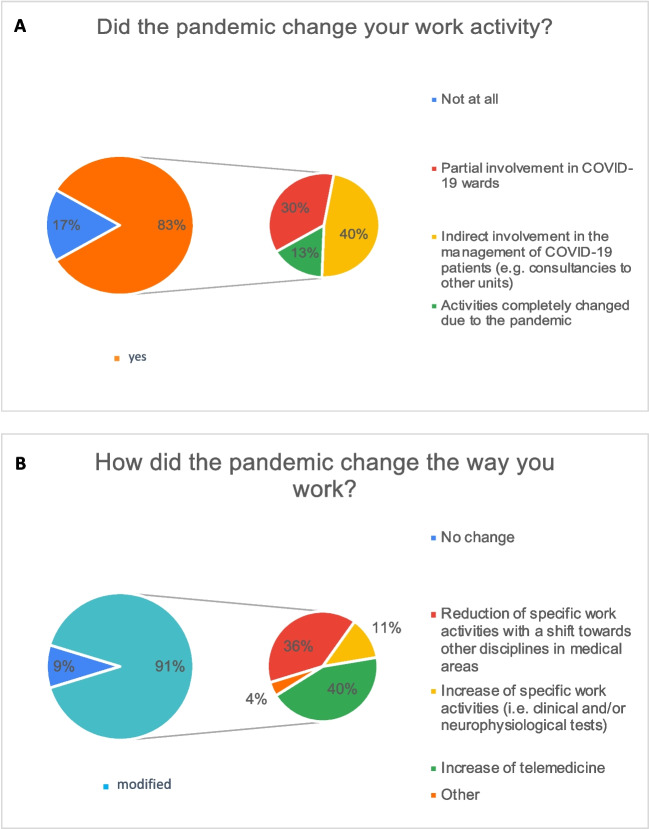
Work changes during the COVID-19 pandemic. Figure shows changes during the pandemic period on work activity (**A**, **B**)

Increased clinical activity was reported by 11.3% of the consultants, due a higher number of visits or neurophysiological examinations required compared to the pre-pandemic situation (Fig. 4A, B).

**Fig. 5:**
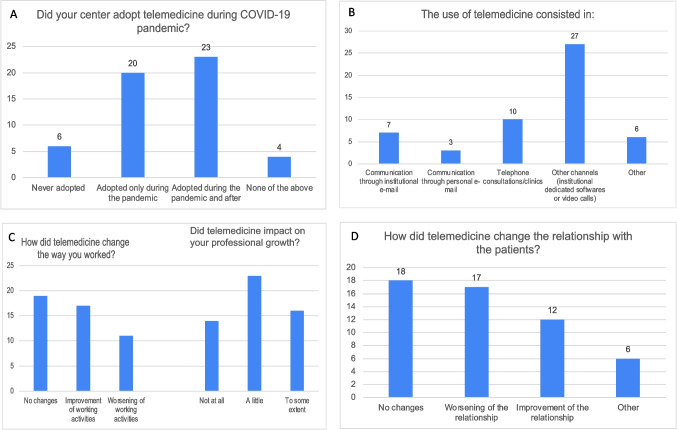
Use of telemedicine during the COVID-19 pandemic. Figure shows the use of telemedicine; in details, the impact of telemedicine on work, professional growth, relationship with patients (**A**, **B**, **C**, **D**)

A considerable number of consultants (39.6%) converted part of their clinical activity from outpatient clinics to telemedicine. Telemedicine consisted mainly of visits by video calls carried out through virtual platforms and specific software (50.9%). Regarding the impact of telemedicine, 32.1% of the clinicians were satisfied because of the improvement of clinical activity, 35.8% perceived no changes, while 20.7% reported a worsening. None (26.4%) or very few (43.4%) participants reported professional growth with the use of telemedicine. Likewise, considering the doctor-patient relationship, 22.6% of participants reported an improvement, 34% no changes, but 32.1% described worsening. The majority of consultant working in Epilepsy Centers (43.4%) used telemedicine during the pandemic period and continue to support the clinical activity with both outpatient and virtual visits, while 37.7% used virtual supports only during the pandemic, and 11.3% of the clinicians never used telemedicine (Fig. 5A, B, C, D). In details, we noticed a tendence of using telemedicine both during the pandemic and after in the South and Center of Italy, while in Islands most of the responders used telemedicine only in pandemic and in the North the respondents were equally divided between those who used telemedicine only during the pandemic and those who continue to use it.

## Discussion

The aim of the presented YES-I survey was to describe the changes observed in the pandemic period on clinicians’ work, educational and research programs, in order to shed lights on several issues faced and consequently improve and prompt the re-organization of education and work of young Italian epileptologists, with direct educational activities proposed by YES-I and LICE.

Our survey highlighted a critical reorganization of epilepsy specific educational programs (70%), clinical activity (81%) and research (54.7%) during the COVID-19 pandemic period for most of the responders. A small proportion of respondents reported no changes in their clinical and educational activities. We can hypothesize that this subset of participants was primarily engaged in research activities, which were only partially affected by the pandemic in comparison to clinical and educational endeavors. Supporting this hypothesis is the presence of PhD students (9%) who participated in our survey, as well as the significant representation of residents (45.3%), who typically allocate part of their training to research activities. It is likely that, during the pandemic, these individuals were predominantly reassigned to University Hospitals rather than to facilities primarily focused on the COVID-19 emergency. In general, the global travel freeze had a negative impact on educational programs and stopped the possibility to gain experience in national and international third-level Epilepsy Centers (70%). Furthermore, trainees, PhD candidates, and early-career clinicians noted a general reorientation of their duties, as reported by 86% of the respondents, towards the care of patients afflicted by SARS-CoV-2. This shift entailed a diminished focus on epilepsy and neurology-related tasks, potentially posing a detrimental influence on the training quality of emerging Italian epileptologists. In line with our observations, a study involved European neurologists and a survey conducted by the young section of the Italian Society of Neurology (SIN) reported the strong reduction of admissions of neurological patients, to cope with the hospitalization of COVID-19 affected patients, hence causing a reduction of educational activities, the shift of work to different expertise and a significant collapse of neurology-specific clinical research and hands-on training [2]; comparable issues have been reported not only within the field of neurology but also across other medical disciplines [10]. All these changes that occurred during the two years of pandemic, considering the duration of post graduate school of just four years in Italy, had a possible negative impact on the quality of training of young Italian epileptologists, not only in the short-term but, a deeper evaluation should be planned in order to evaluate the long-term consequences of this educational hindrance during the pandemic. As a result, in order to mitigate the reduction in educational avenues, there has been a surge in online educational resources, courses, and conferences. Within this context, the International League Against Epilepsy (ILAE), LICE (Italian League Against Epilepsy) and its task forces, such as the Education and Career Development Task Force of YES-I (https://www.lice.it/LICE_ita/YES-I/YES_I.php), have introduced an expanding number of opportunities aimed at enhancing scientific knowledge in the field of epilepsy. These include webinars, podcasts, and e-learning courses, with different e-learning levels, and intermediate and final assessment to evaluate learning and effectiveness, highlighting the need of changing learning strategies requested and preferred by young students [10, 11]. Beyond the obvious advantages in terms of cost reduction, easier organization of work activities, time efficiency given the reduction in travel times, better accessibility and flexibility, there are important global values to consider, including the reduction of environmental impact of traveling, increased collaboration between centers and communication among distant research groups. However, a considerable number of our responders (50.9%) considered the global level of online education under the standard. Interestingly, the online webinars organized by the YES-I were considered as a high-quality training experience by 58.5% of the participants. The favorable response may be attributed to the engagement of young trainees, who are well-acquainted with the educational requirements and are capable of proposing innovative teaching methods. Their involvement has the potential to enhance the effectiveness and quality of educational programs, webinars, and conferences. We believe that our survey shed light on a prerequisite for the future of e-learning not only among young epileptologists, i.e. the quality of education. Future proposals, aimed at ensure high-quality of education, also with virtual methods, in order to reduce long-term consequences of the possible under standard quality education faced during the pandemic and bridge centers inequalities in educational trainings among different Italian regions, should encompass the integration of webinars, e-learning modules and platforms, with different level of proficiency that incorporate pre- and post-assessment measures, at each step, to assess the efficacy of educational initiatives. These measures should also evaluate user satisfaction and consider the inclusion of more interactive instruction methods, including engagement with tutors and multimedia resources such as quizzes and exercises. Problem-solving learning strategies, which have demonstrated their effectiveness in enhancing the learning experience and boosting motivation, should also be considered [12–14]. Furthermore, a greater involvement of young trainers, who know their educational needs and can suggest innovative ways of teaching, could help in improving effectiveness and quality of educational programs, webinars and conferences.

The reduction of hospital admissions and face-to-face outpatient clinics facilitated the increase of remote consultations. Several studies have highlighted the need to maintain a close contact with patients afflicted by epilepsy throughout the pandemic, owing to an increase in seizures and psychological issues stemming from neurological complications arising from infections or suboptimal clinical management of chronic conditions [3, 15]. Although the usefulness of telemedicine was already described years before the pandemic period, particularly for epilepsy patients living in rural areas [16], only the COVID-19 lockdown allowed the dissemination of telemedicine. Virtual medicine offers many advantages, both for healthcare providers and patients, such as the possibility to reach people distant from medical facilities, reduced travel time for patients, avoid misunderstanding in making dosage drugs adjustments, and the ease of sharing information with tertiary epilepsy centers in complex cases [17]. Despite all these advantages only a small number of our responders expressed satisfaction with telemedicine, and only a few reported an amelioration of clinical activity. Interestingly, less than 50% of the answerers employed telemedicine during the pandemic period, and now continue to use virtual systems in clinical practice. The reason for this limited diffusion could be attributed to the inability to conduct a complete neurological evaluation, and a more difficult doctor-patient interaction compared with face-to-face outpatient visits, particularly regarding patients with intellectual disability or autism spectrum disorders [18]. The same disappointment was reported in other studies, where workload associated with telemedicine organization was the principal deterrent to its post-pandemic adoption [19, 20]. A negative impact in doctor-patient relationship was indeed reported by a notable number of clinicians in our survey (32.1%). Without direct investigations concerning the reason of such worsening, we can speculate that this was possibly due to the wide use of email and phone calls without video reported by many clinicians (35.8%) instead of the use of software enabling videocalls, which may have contributed to making communication and relationship more difficult compared the face-to-face visits. On the other hand, several studies remarked both patients and parents’ satisfaction with telemedicine due to reduced time for travel, costs and the possibility to have an easy contact with the clinician also for the patients without driving license due to their epilepsy [21, 22]. Face-to face visits were still considered more appropriate and preferred for the first consultation and for patients with drug resistant epilepsy [22]. Further studies should be conducted to investigate the factors contributing to the dissatisfaction with telemedicine as perceived by certain clinicians and patients. Additionally, these studies should explore the psychological impact of this virtual communication method to enhance the effectiveness of telemedicine and improve doctor-patient relationships. Our studies underlined some differences in Italian regions, with Center and South with where telemedicine is spreading and North and Islands where clinicians are still divided in its use, therefore, further investigations are needed in order to evaluate in which centers telemedicine was abandoned, which were the underlying causes in order to find possible solutions in improving the use and its diffusion in the future. The goal of future medicine will be maintaining a high quality of medical care not only through traditional patients’ management but also by using virtual methods. In this context, the ILAE Telemedicine Task Force suggested recommendations to improve the quality and effectiveness of virtual visits including videoconferencing in order to create a good relationship with the patients and family, questionnaires to describe seizures and a self-questionnaire to investigate comorbidities, instruction of patients and family members when outpatient consultation or emergency department is needed [23]. Moreover, future studies should point out possible psychological impact of use of telemedicine instead of classical outpatient visits both on patients and healthcare workers.

During the pandemic era, our investigation and existing literature reveal to some extent impediment to research activities [2, 4], with a notable de-prioritization of other research endeavors involving patients. Nevertheless, the virtual modality has also made significant contributions in this domain by fostering research momentum. Specifically, it has facilitated the connection of geographically distant research groups, expanded the potential for multicenter collaborations, enabled data sharing, streamlined information exchange among both primary and tertiary epilepsy centers in cases of patient management challenges, and promoted the exchange of knowledge that was previously underemphasized [4].

While this study makes significant contributions to assess the impact of the pandemic on the training and work of young epileptologists and ideas for improving future, it has some limitations: the selection bias due to lack of information about the specific centers where responders work presents a limitation in understanding the regional disparities and challenges faced by different centers during and after the pandemic; incorporating this information in subsequent studies, will allowed to analyze the critical issues faced by each center in order to create a more homogeneous healthcare landscape, and address inequalities; the limited sample size which may not adequately represent the comprehensive perspectives of the entire population of Italian epileptologists. Although the study was aimed to provide subsequent educational, research and work opportunities specifically for young epileptologists (< 40 years) and not focuses on senior Italian epileptologists, understanding the changes perceived by also senior epileptologists in future studies will be useful in potential global improvement measures for all Italian epileptologists, not only during their initial training but also throughout their career path.

## Conclusion

Our survey highlighted the negative pandemic impact of the COVID-19 pandemic on training, research and clinical activity in the epilepsy field and the need of a remodulation in the different epilepsy areas.

Despite the growing of evidence demonstrating the feasibility and effectiveness of remote systems in clinical, research, and work-related activities, as well as in e-health and e-learning, it is notable that in Italy, particularly among younger generations, these innovations are perceived more as an obstacle than an advancement. Our survey had the merit of shading light on the critical aspects, of virtual communication system, first of all the acceptance, but also the possible long-term consequences of the reduction of hands-on training and inpatient visits; however, it highlighted the need for robust evaluation methods to ensure virtual educational quality and effectiveness and to improve the quality doctor-patient relationships using telemedicine. Our work suggested possible actionable steps, some of them already implemented by The Education and Career Development Task Force of the Young Epilepsy Section-Italy (YES-I), in order to improve virtual education and telemedicine.

Future studies conducted on a larger sample of Italian epileptologists and aimed to investigate patients’ and clinicians’ perspectives on virtual systems are needed in order to improve their quality, make them more functional, efficacious and so to be preferred in clinical activity, research and learning in the coming years, with a direct future proposal of YES-I LICE and LICE.

## Data Availability

Anonymized data that support the findings of this study are available from the corresponding author (B.N.) on reasonable request.

## Abbreviations

COVID-19: Coronavirus Disease 19
ILAE-YES: Young Epilepsy Section of International League Against Epilepsy
LICE: Italian League Against Epilepsy
SARS-CoV-2: Severe Acute Respiratory Syndrome Coronavirus 2
YES-I: Young Epilepsy Section-Italian
